# Different Radiological Criteria for Early Tumor Response Evaluation in Patients With Unresectable Hepatocellular Carcinoma Treated With Anti-PD-1 Antibody Plus Bevacizumab

**DOI:** 10.3389/fonc.2022.848129

**Published:** 2022-04-19

**Authors:** Ying Xu, Yi Yang, Lu Li, Aiping Zhou, Hongmei Zhang, Feng Ye, Wen Zhang, Hong Zhao, Xinming Zhao

**Affiliations:** ^1^ Department of Diagnostic Radiology, National Cancer Center/National Clinical Research Center for Cancer/Cancer Hospital, Chinese Academy of Medical Sciences and Peking Union Medical College, Beijing, China; ^2^ Department of Hepatobiliary Surgery, National Cancer Center/National Clinical Research Center for Cancer/Cancer Hospital, Chinese Academy of Medical Sciences and Peking Union Medical College, Beijing, China; ^3^ Key Laboratory of Gene Editing Screening and Research and Development (R&D) of Digestive System Tumor Drugs, Chinese Academy of Medical Sciences and Peking Union Medical College, Beijing, China; ^4^ Department of Medical Oncology, National Cancer Center/National Clinical Research Center for Cancer/Cancer Hospital, Chinese Academy of Medical Sciences and Peking Union Medical College, Beijing, China

**Keywords:** hepatocellular carcinoma, targeted therapy, immunotherapy, tumor response, overall survival

## Abstract

**Purpose:**

We aimed to compare different radiological criteria in evaluating the early tumor response of patients with unresectable hepatocellular carcinoma (uHCC) treated with an anti-programmed cell death protein 1 (PD-1) antibody plus bevacizumab.

**Method:**

From October 2018 to January 2020, 58 patients [49 (84.5%) men, age = 55.2 ± 10.6 years] receiving both anti-PD-1 antibody and bevacizumab were retrospectively included. Pre- and the first posttreatment contrast-enhanced computed tomography (CE-CT) scans were performed in all patients. The Response Evaluation Criteria in Solid Tumors, version 1.1 (RECIST 1.1), modified RECIST (mRECIST), Choi, and the revised Choi (rChoi) criteria were applied to evaluate tumor response. The endpoint event was defined as overall survival (OS).

**Results:**

Six (10.3%), 9 (15.5%), 30 (51.7%), and 12 (20.7%) patients were diagnosed as responders by RECIST 1.1, mRECIST, Choi, and rChoi, respectively. The RECIST 1.1 and mRECIST criteria failed to correlate the evaluation categories with OS (*p* = 0.130 and 0.253, respectively), while both Choi and rChoi significantly correlated with OS (*p* = 0.002 and 0.006, respectively). Among the four criteria, only those patients identified as responders by Choi (*p* = 0.0005) and rChoi (*p* = 0.005) showed significantly better OS than the non-responders. The cumulative 1- and 2-year OS rates by Choi were 93.3% and 79.8% in responders and 69.3% and 30.3% in non-responders, respectively; these rates were 100.0% and 100.0% in responders and 74.9% and 43.1% in non-responders by rChoi, respectively.

**Conclusions:**

The evaluation of early tumor response using Choi and rChoi instead of RECIST 1.1 and mRECIST significantly correlated with the OS of patients with uHCC treated with an anti-PD-1 antibody plus bevacizumab. Moreover, patients identified as responders by Choi and rChoi showed significantly better OS than the non-responders.

## Highlights

(1) This is the first study to perform early tumor response evaluation using different radiological criteria and correlate with OS in patients with uHCC treated with an anti-PD-1 antibody plus anti-angiogenesis targeted therapy.
(2) Evaluation of early tumor response using Choi and rChoi instead of RECIST 1.1 and mRECIST significantly correlated with the OS of patients with uHCC treated with an anti-PD-1 antibody plus anti-angiogenesis targeted therapy.
(3) Choi and rChoi showed promise in identifying early tumor response, and patients identified as responders using these two criteria showed significantly better OS than the non-responders.

## Introduction

Currently, liver cancer is estimated to be the sixth most common cancer worldwide and the third leading cause of cancer-related deaths (1). Hepatocellular carcinoma (HCC) accounts for about 90% of liver cancer (1). However, a majority of patients were diagnosed as unresectable HCC (uHCC), with unfavorable prognoses (2, 3).

The combination of an immune checkpoint inhibitor [anti-programmed cell death protein 1 (PD-1) and anti-programmed cell death ligand-1 (PD-L1) antibodies] and anti-angiogenesis targeted therapy has been proven effective for patients with uHCC. The IMbrave150 study indicated that atezolizumab (an anti-PD-L1 antibody) combined with bevacizumab (anti-angiogenesis targeted therapy) was beneficial to overall survival (OS) and progression-free survival (PFS) compared with sorafenib in patients with uHCC [hazard ratio (HR) for death of atezolizumab–bevacizumab *vs.* sorafinib = 0.58, *p* < 0.001; median PFS = 6.8 *vs.* 4.3 months, *p* < 0.001] (4). In addition, the ORIENT-32 study revealed that patients with uHCC treated with sintilimab (an anti-PD-1 antibody) combined with bevacizumab showed prolonged OS and PFS compared with patients given sorafenib (median PFS = 4.6 *vs.* 2.8 months, *p* < 0.0001; median OS = not reached *vs.* 10.4 months, *p* < 0.0001) (5). To date, more and more combination therapies of an anti-PD-1 antibody plus anti-angiogenesis targeted therapy have been investigated and approved as first-line systemic therapy for patients with advanced HCC (6, 7). Consequently, precise evaluation of the tumor response brought about by systemic therapies, especially combined anti-PD-1 antibody plus anti-angiogenesis targeted therapy, is crucial for clinical decision-making.

Response Evaluation Criteria in Solid Tumors, version 1.1 (RECIST 1.1), has been established for tumor response evaluation in solid tumors (8). However, based on the unidimensional diameter, RECIST 1.1 may underestimate the tumor response, and it has been reported to have poor correlations with the clinical outcomes of patients with HCC after targeted therapies (9, 10). Tumor response caused by anti-angiogenesis targeted therapy usually occurs with minimal size shrinkage, which is generally insufficient to meet the RECIST-defined response threshold (30%). The reduction in “viable tumor”, considering treatment-related intratumoral necrosis, was proposed by the modified RECIST (mRECIST) criteria to overcome the disadvantage of RECIST 1.1 (11, 12). The Choi criteria (incorporating tumor size and attenuation), which was initially proposed for gastrointestinal stromal tumors (GISTs) (13), has also been applied for tumor response in patients with HCC (9, 14). The revised Choi (rChoi) was modified on the basis of Choi and was first described in the study of Thian et al. to evaluate tumor response in metastatic renal cell carcinoma (mRCC) treated with sunitinib (15). The rChoi defined partial response (PR) as both a 10% decrease in the tumor size and a 15% decrease in the tumor density of the target lesions, while Choi defined PR as either deduction of the tumor size or density.

Currently, there is a lack of studies on the radiological criteria established for tumor response in patients with uHCC treated with an anti-PD-1 antibody plus anti-angiogenesis targeted therapy. In this study, we aimed to assess early tumor response using different radiological criteria (RECIST 1.1, mRECIST, Choi, and rChoi) and to correlate the evaluation categories with the OS of patients with uHCC treated with an anti-PD-1 antibody plus bevacizumab.

## Materials and Methods

### Patient Selection

From October 2018 to January 2020, 78 patients 18 years of age or older with uHCC who received treatment of an anti-PD-1 antibody plus bevacizumab were retrospectively included at our institution. The study protocol had been reviewed and approved by the Ethics Committee of our institution (18-126/1704). Written informed consent for therapy was obtained from each patient. An *a priori* study design was not required because of the descriptive and pragmatic nature of the study.

**Figure 1:**
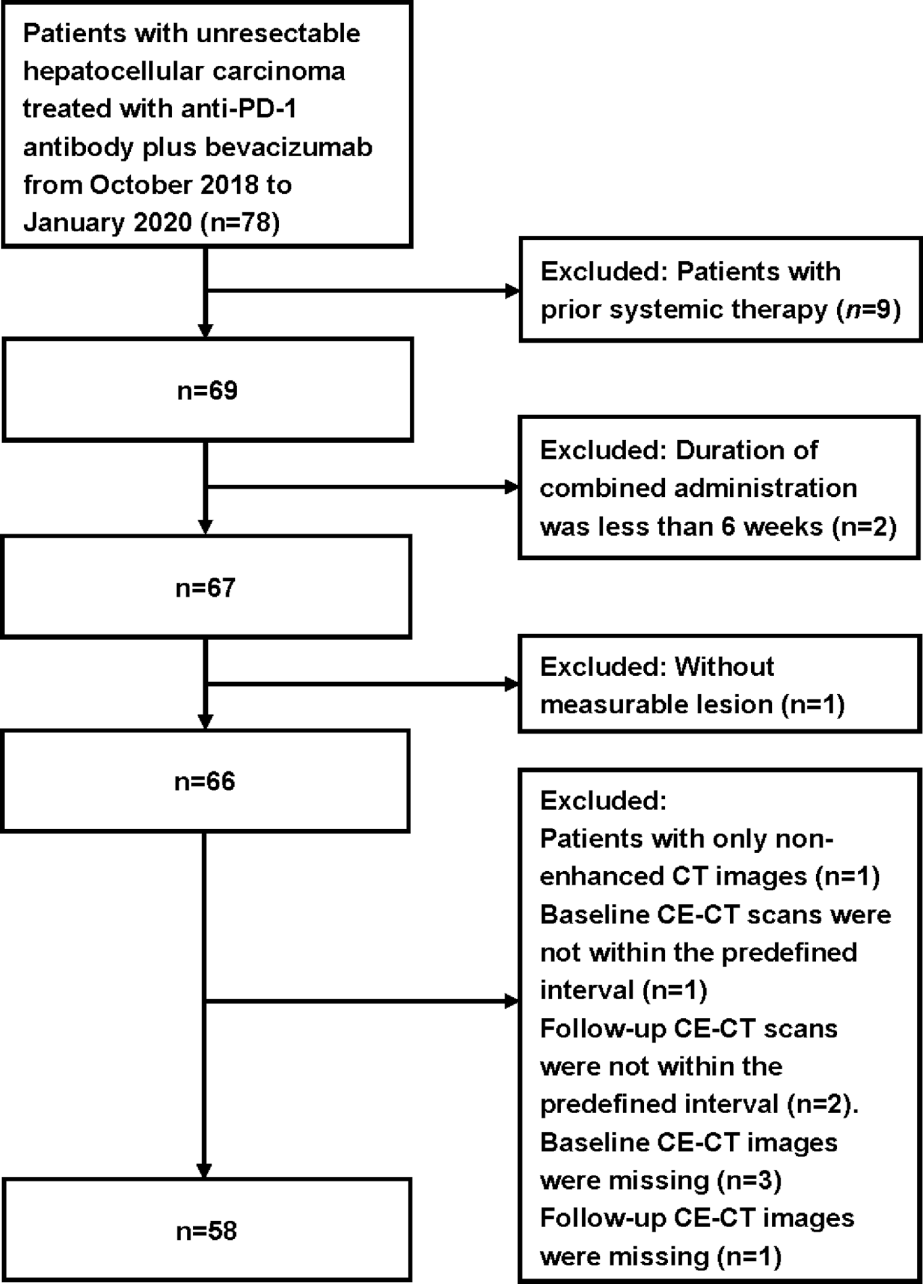
Flowchart of patient selection.

The inclusion criteria were as follows: 1) patients with uHCC confirmed by histology/cytology; 2) patients without previous systemic therapy for liver cancer, unsuitable for transarterial chemoembolization (TACE), or had TACE treatment failure; 3) patients who received a minimal cumulative duration of 6 weeks of anti-PD-1 antibody plus bevacizumab; 4) Barcelona Clinic Liver Cancer (BCLC) C or BCLC B, Eastern Cooperative Oncology Group Performance Status (ECOG PS) 0 or 1, and Child–Pugh score ≤7 points, and adequate hematological and organ function; 5) patients with the contrast-enhanced computed-tomography (CE-CT) of the thorax, abdomen, and pelvis performed within 1 month before treatment as baseline and a follow-up CE-CT scan after a 6-week treatment; and 6) at least one measurable lesion as per RECIST 1.1. The exclusion criteria were: 1) baseline or follow-up CE-CT images missing or obtained without contrast agent; 2) baseline or follow-up CE-CT scans not within the predefined interval; and 3) without measurable lesion. The flowchart for patient selection is shown in **Figure 1**.

### Clinical Therapy

Sintilimab (*n* = 48; TYVYT^®^, Innovent, Jiangsu, China), pembrolizumab (n = 7; Keytruda® Merck, New Jersey, USA), and camrelizumab (*n* = 3; AiRuiKa^®^, Hengrui, Jiangsu, China) were given intravenously at 200 mg over 60 min, followed by the optimal dose of bevacizumab (*n* = 58; Avastin®, Roche, Basel, Switzerland) given intravenously over 90 min every 3 weeks, according to the individual condition assessed by clinicians. Dose alterations or interruptions were performed in consideration of individual safety according to the decision made by the clinicians (WZ and AZ, with 20 and 25 years of experience, respectively, in medical oncology). Combination therapy was maintained until unacceptable toxicity, progressive disease (PD), withdrawal of informed consent, death, or other circumstances that required termination of treatment, whichever occurred first.

### CT Scan Protocols

CE-CT imaging of the thorax, abdomen, and pelvis at baseline and follow-up of all patients was performed with a 64-detector row scanner (GE Optima 660 or Discovery 750; General Electric Medical System, Chicago, IL, USA). Iobitridol (320 mg/ml iodine, iodixanol injection; Beijing Beilu Pharmaceutical CO., LTD., Beijing, China) was intravenously injected at a dose of 1.5 ml/kg using a power injector at a flow rate of 3.0 ml/s. CE-CT images of the abdomen on arterial, portal venous, and equilibrium phases were obtained 35, 65, and 150 s after contrast agent administration, respectively (tube voltage, 120 kVp; auto mA settings; pitch, 1.375; rotation time, 0.5 s; thickness, 5 mm).

### Identification of Target Lesions and Radiological Evaluation

**Figure 2:**
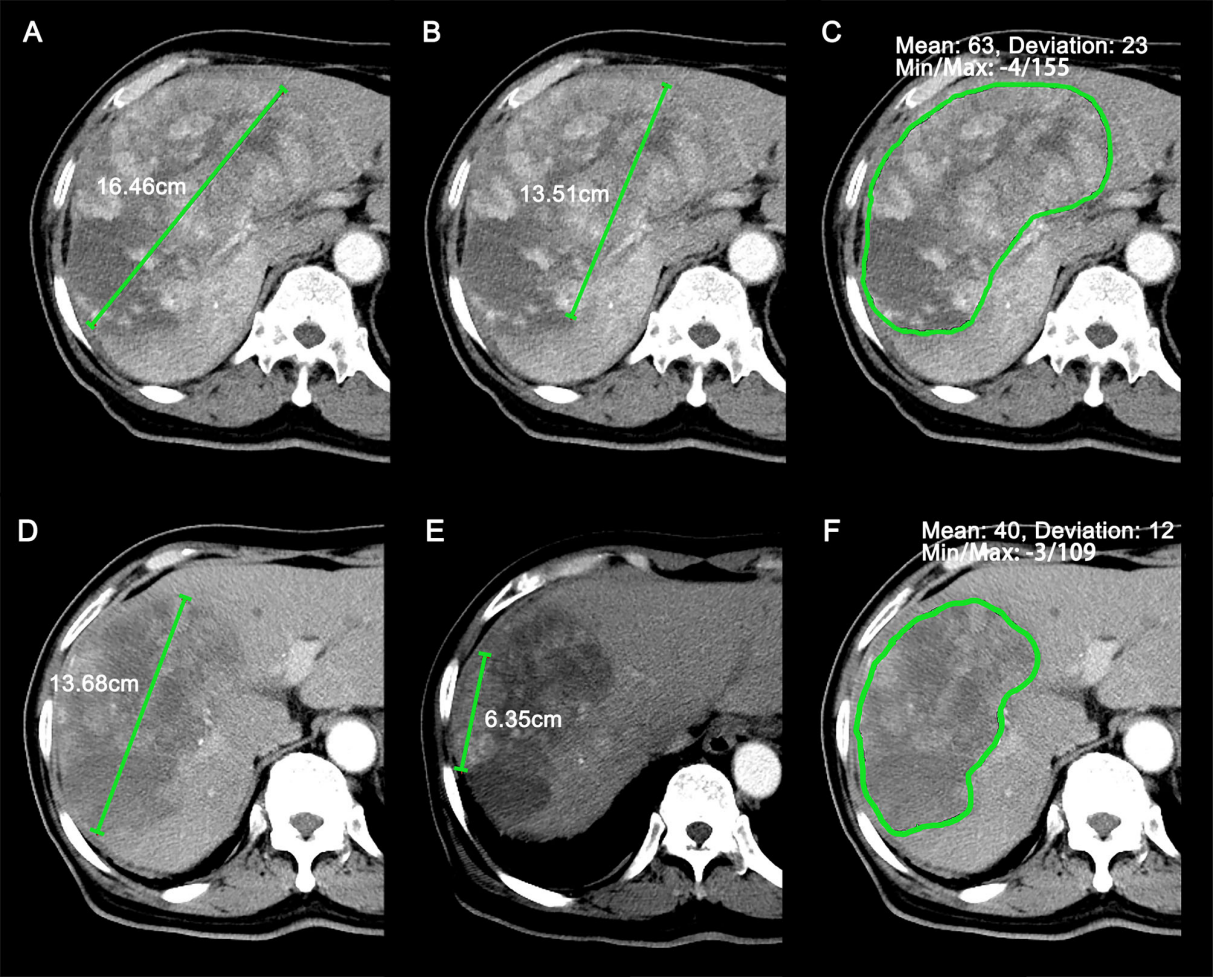
Representative case (69-year-old male patient) of target lesions measured at baseline and the first follow-up scan. **(A–C)** Sum of longest diameters (SLDs) **(A)**, SLDs of viable (enhancing) target lesions **(B)**, and average attenuation of the target lesions **(C)** at baseline CT images. **(D–F)** SLDs **(D)**, SLDs of viable (enhancing) target lesions **(E)**, and average attenuation of the target lesions **(F)** at the first follow-up CT images. The patient was identified as stable disease (SD), partial response (PR), PR, and PR by RECIST 1.1, modified RECIST (mRECIST), Choi, and revised Choi (rChoi), respectively.

On baseline CE-CT images, the target lesions should be at least 1.0 cm for the longest diameter according to RECIST 1.1. For each patient, a maximum of two lesions per organ and five lesions in total were selected (8). From the baseline and the first follow-up CE-CT images, we measured the sum of the longest diameters (SLDs) of target lesions using RECIST 1.1, the SLDs of viable (enhancing) target lesions using mRECIST, and the average density of the target lesions using the Choi and rChoi criteria (9, 14, 16) (**Figure 2** and **Table 1**). For Choi and rChoi, freehand-drawn regions of interest (ROIs) were delineated around the contour of the target lesions. The density of the ROIs was presented with attenuation values measured in Hounsfield unit (HU). When more than one lesion was selected as target, the average density of all the target lesions was calculated. All the measurements were performed at the arterial phase instead of the portal phase due to HCC being abundant with blood supply and showing obvious enhancement in the arterial phase, as previously reported (9, 14, 16).

**Table 1:** RECIST 1.1, modified RECIST (mRECIST), Choi, and revised Choi (rChoi) criteria.

Criteria	RECIST 1.1	mRECIST	Choi	rChoi
CR	Disappearance of all target lesions. No new lesions	Disappearance of any intratumoral arterial enhancement in all target lesions	Disappearance of all target lesions. No new lesions	Disappearance of all target lesions. No new lesions
PR	≥30% decrease in the tumor size of target lesions. No new lesions	≥30% decrease in the tumor size of viable (enhancement in the arterial phase) target lesions, taking as reference the baseline target lesions	≥10% decrease in the tumor size of target lesions or ≥15% decrease in the tumor density of target lesions. No new lesions	≥10% decrease in the tumor size, ≥15% decrease in the tumor density of the target lesion or no lesions suitable for density analysis, and ≥30% decrease in the tumor size of target lesions
SD	Neither PR nor PD	Neither PR nor PD	Neither PR nor PD	Neither PR nor PD
PD	≥20% increase in the tumor size of target lesions and an absolute increase of at least 5 mm. New lesions	≥20% increase in the tumor size of viable (enhancing) target lesions, taking as reference the smallest sum of the diameters of the target lesions since treatment started. New lesions	≥10% increase in the tumor size and does not meet the PR criteria by tumor density (HU) on CE-CT scan. New lesions	≥10% increase in the tumor size and does not meet the PR criteria by tumor density (HU) on CE-CT scan. New lesions

CR, complete response; PR, partial response; SD, stable disease; PD, progressive disease; CE-CT, contrast-enhanced computed tomography.

Radiological evaluation was performed by two independent radiologists (LL and YX, with 10 and 5 years of experience, respectively, in abdominal radiology) at baseline and the first follow-up CE-CT scans. The two radiologists selected the target and non-target lesions and assessed each patient independently, blinded to the other’s evaluation results. Discrepancy between the two radiologists was adjudicated by a third senior radiologist (FY, with 18 years of experience in abdominal radiology) to reach a consensus. All three radiologists were blinded to the patients’ clinical data and outcome. Responders included patients with complete response (CR) or PR, and non-responders included patients with stable disease (SD) or PD.

### Endpoint of Study

The endpoint of this study was OS, which was calculated from the date of initiation of the anti-PD-1 antibody plus bevacizumab until death by any cause or the last follow-up. The last follow-up was completed on October 6, 2021.

### Statistical Analysis

Categorical variables were presented as frequencies with percentages, and continuous variables were presented as median with interquartile range (IQR). The OS curves of early tumor response evaluation using the different criteria were prepared using the Kaplan–Meier method with the log-rank test. Reverse Kaplan–Meier estimate of OS was used to calculate the time of follow-up (17). A *p-*value <0.05 was considered as statistically significant. Variability of tumor response between two radiologists was presented by weighted *k* statistics. The weighted *k* coefficients were stratified as follows: 0.81–1.00, almost perfect; 0.61–0.80, substantial; 0.41–0.60, moderate; 0.21–0.40, fair; ≤0.20, poor (18). All analyses were performed using SPSS 22.0 (SPSS Inc., Chicago, IL, USA).

## Results

### Baseline Characteristics

Seventy-eight patients without prior systemic therapy received a minimal cumulative duration of 6 weeks of combined treatment. Fifty-eight patients with 117 target lesions were eligible for the present study. Nine patients were excluded due to prior systemic therapy and two patients excluded due to inadequate duration of the combined administration. Eight patients were excluded due to missing CE-CT images or out of the predefined interval. One patient was excluded due to lacking measurable lesions. The median therapy duration was 5.8 months (IQR = 2.4–11.4). The median time from therapy initiation to first evaluation was 1.3 months (IQR = 1.2–1.5). The baseline characteristics of the included patients are shown in **Table 2**.

**Table 2:** Patient and baseline characteristics.

Characteristics	Total patients (*n* = 58), mean ± SD/median (range)/*n* (%)
Age (years)	55.2 ± 10.6
Male sex	49 (84.5)
ECOG PS	
0	23 (39.7)
1	35 (60.3)
Macrovascular invasion	
Yes	21 (36.2)
No	37 (63.8)
Extrahepatic disease	
Yes	34 (58.6)
No	24 (41.4)
Site of target lesion	
Liver	50 (86.2)
Lung	7 (12.1)
Lymph node	16 (27.6)
Peritoneum	8 (13.8)
Bone	2 (3.4)
Ovary	1 (1.7)
Adrenal gland	1 (1.7)
Baseline α-fetoprotein ≥200 ng/ml	28 (48.3)
Child–Pugh class	
A	57 (98.3)
B	1 (1.7)
BCLC stage	
B (intermediate)	13 (22.4)
C (advanced)	45 (77.6)
Liver cirrhosis (investigator assessed)	
Yes	39 (67.2)
No	19 (32.8)
Etiology of HCC: hepatitis B virus	
Yes	56 (96.6)
No	2 (3.4)
No. of target lesions	
1	19 (32.7)
2	25 (43.1)
3	11 (19.0)
5	3 (5.2)
First-line therapy	58 (100.0)
Duration of combined treatment (months)	5.8 (2.4–11.4)
Time between initiation and first evaluation (months)	1.3 (1.2–1.5)

ECOG PS, Eastern Cooperative Oncology Group Performance Status; BCLC, Barcelona Clinic Liver Cancer; HCC, hepatocellular carcinoma.

### Tumor Response Evaluation

**Figure 3:**
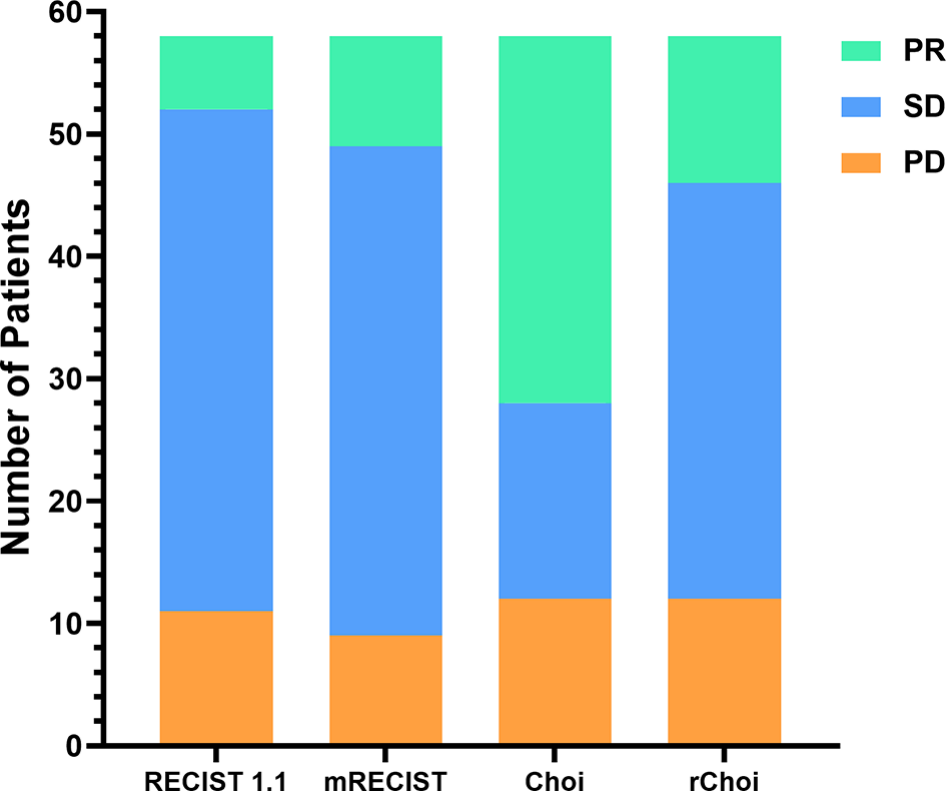
Comparisons of the evaluations by the RECIST 1.1, modified RECIST (mRECIST), Choi, and revised Choi (rChoi) criteria.

**Figure 4:**
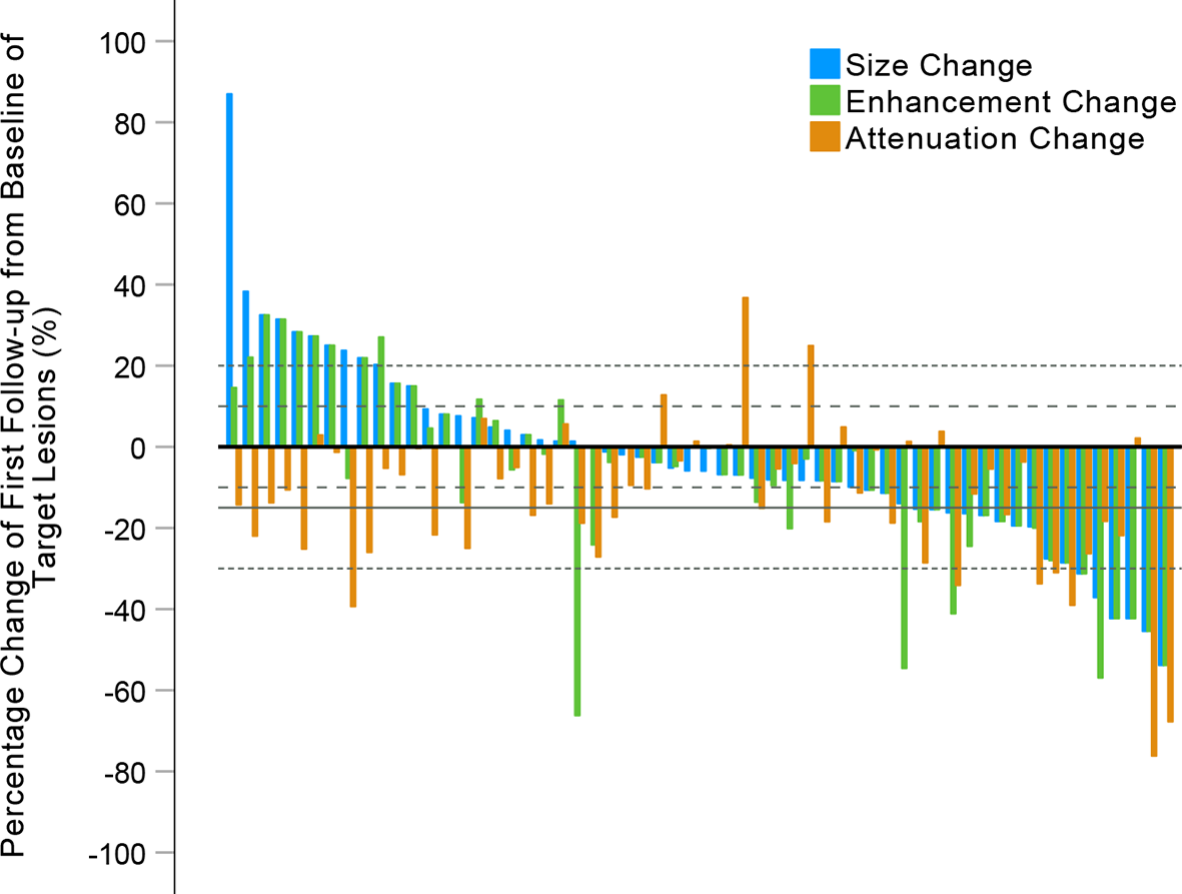
Waterfall plots summarizing the maximum percent changes from baseline in the sum of longest diameters (SLDs), the SLDs of viable (enhancing) target lesions, and the attenuation of target lesions at the first follow-up CE-CT scan. The *three adjacent bars* in each group represent one patient. *Thin dashed lines* indicate the size thresholds for the RECIST 1.1 and modified RECIST (mRECIST) criteria, and *thick dashed lines* the size thresholds for the Choi and revised Choi (rChoi) criteria. *Solid line* indicates the attenuation threshold for the Choi and rChoi criteria.

The performance of the four criteria in the evaluation of tumor response is shown in **Figure 3**. The changes of the SLDs from baseline to the first follow-up CE-CT, the SLDs of viable (enhancing) areas, and the average density of all the target lesions for each patient are presented in **Figure 4**.

According to RECIST 1.1 and mRECIST, the majority of patients [41 (70.7%) and 40 (69.0%), respectively] were defined as SD, while fewer patients [16 (27.6%) and 34 (58.6%), respectively] were defined as SD by Choi and rChoi. On the contrary, more patients were defined as PR by Choi and rChoi [30 (51.7%) and 12 (20.7%), respectively] than by RECIST 1.1 and mRECIST [6 (10.3%) and 9 (15.5%), respectively]. According to RECIST 1.1, mRECIST, Choi, and rChoi, 11 (19.0%), 9 (15.5%), 12 (20.7%), and 12 (20.7%) patients, respectively, were defined as PD.

### Survival Analysis

**Figure 5:**
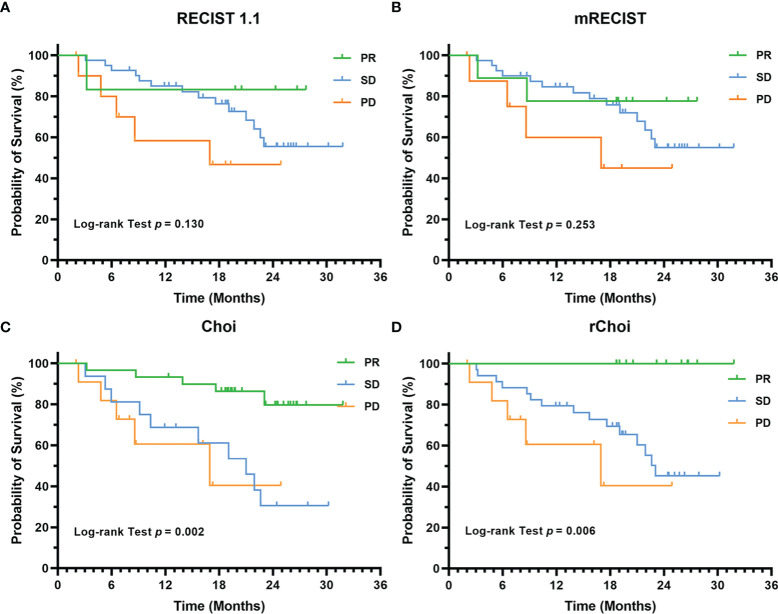
Kaplan–Meier curves for the overall survival (OS) of 58 patients with unresectable hepatocellular carcinoma (uHCC) as categorized into partial response (PR), stable disease (SD), and progressive disease (PD) by the RECIST 1.1 **(A)**, modified RECIST (mRECIST) **(B)**, Choi **(C)**, and revised Choi (rChoi) **(D)** criteria.

Twenty (34.5%) patients died during a median follow-up duration of 23.2 months (95% CI = 18.1–28.3 months). The cumulative 1- and 2-year OS rates were 80.3% and 56.2%, respectively. RECIST 1.1 and mRECIST failed to correlate the evaluation categories with OS (*p* = 0.130 and 0.253, respectively), while both Choi and rChoi significantly correlated with OS (*p* = 0.002 and 0.006, respectively) (**Figure 5**).

**Figure 6:**
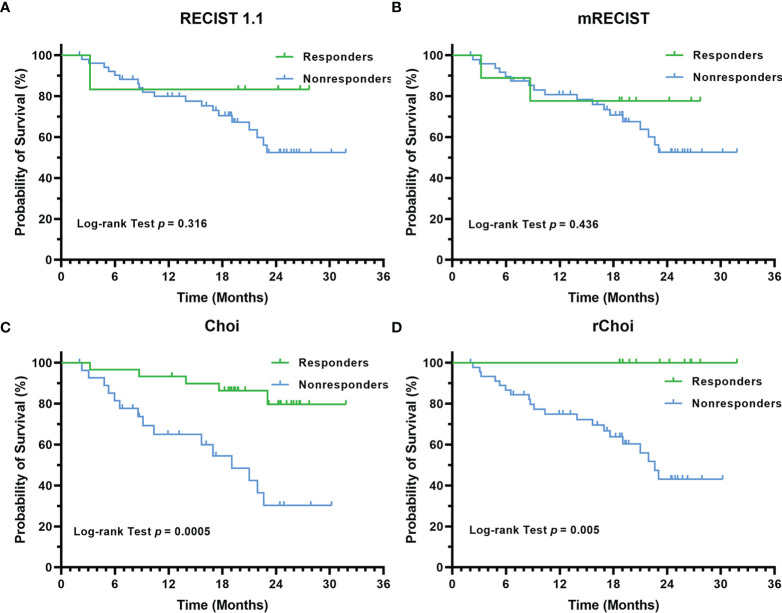
Kaplan–Meier curves for the overall survival (OS) of 58 patients with unresectable hepatocellular carcinoma (uHCC) as categorized into responders and non-responders by the RECIST 1.1 **(A)**, modified RECIST (mRECIST) **(B)**, Choi **(C)**, and revised Choi (rChoi) **(D)** criteria.

Among the four criteria, only those patients identified as responders by Choi (*p* = 0.0005) and rChoi (*p* = 0.005) showed significantly better OS than the non-responders (**Figure 6**). The cumulative 1- and 2-year OS rates by Choi were 93.3% and 79.8% in responders and 69.3% and 30.3% in non-responders, respectively; these rates were 100.0% and 100.0% in responders and 74.9% and 43.1% in non-responders by rChoi, respectively.

### Inter-Reader Variability

According to the results of the blinded evaluation, the differences between the two radiologists in tumor response evaluation using the four criteria were acceptable. The inter-reader variability for all criteria is summarized in **Table 3**. The inter-radiologist agreements for RECIST 1.1, mRECIST, Choi, and rChoi were 96.6%, 94.8%, 98.3%, and 100.0%, respectively. The weighted *k* coefficients for the RECIST 1.1, mRECIST, Choi, and rChoi criteria were 0.93 (0.51–1.03), 0.90 (0.78–1.01), 0.97 (0.92–1.03), and 1.00 (1.00–1.00), respectively.

**Table 3:** Response rates according to the evaluation criteria for two independent radiologists with inter-reader agreement (*n* = 58).

Tumor response, *n* (%)	RECIST 1.1		mRECIST		Choi		rChoi	
	R1	R2	R1	R2	R1	R2	R1	R2
CR	0 (0)	0 (0)	0 (0)	0 (0)	0 (0)	0 (0)	0 (0)	0 (0)
PR	6 (10.3)	7 (12.1)	9 (15.5)	11 (19.0)	30 (51.7)	29 (50.0)	12 (20.7)	12 (20.7)
SD	41 (70.7)	39 (67.2)	40 (69.0)	37 (63.8)	16 (27.6)	17 (29.3)	34 (58.6)	34 (58.6)
PD	11 (19.0)	12 (20.7)	9 (15.5)	10 (17.2)	12 (20.7)	12 (20.7)	12 (20.7)	12 (20.7)
Inter-reader agreement, *n* (%)	56 (96.6)		55 (94.8)		57 (98.3)		58 (100.0)	
Weighted *k* (95% CI)	0.93 (0.51–1.03)		0.90 (0.78–1.01)		0.97 (0.92–1.03)		1.00 (1.00–1.00)	

R1, radiologist 1; R2, radiologist 2; CR, complete response; PR, partial response; SD, stable disease; PD, progressive disease.

## Discussion

The combination of immunotherapy with targeted therapy has currently been the trend of systemic therapy for patients with uHCC (1). However, new therapies posed challenges for the evaluation of tumor response. Our study attempted to compare the performance of different radiological criteria (RECIST 1.1, mRECIST, Choi, and rChoi) in evaluating early tumor response in this target population and to correlate with OS. It was demonstrated that evaluation of early tumor response by RECIST 1.1 and mRECIST failed to correlate with the OS of patients with uHCC treated with an anti-PD-1 antibody plus bevacizumab, while the evaluation using the Choi and rChoi criteria significantly correlated with OS. Moreover, the responders identified using the Choi and rChoi criteria at an early stage showed significantly better OS than the non-responders.

Our previous study reported the different criteria used in tumor response evaluation in patients with metastatic colorectal cancer (mCRC) treated with the combination of regorafenib and an anti-PD-1 antibody (19). This study revealed that the RECIST 1.1, Choi, and rChoi criteria could all identify the survival benefit from treatment with regorafenib plus an anti-PD-1 antibody in mCRC patients. Besides, those identified as responders seemed to show better OS than the non-responders according to Choi, although statistical significance was not reached owing to the limited sample size (*p* = 0.262). In the present study, evaluation of early tumor response using Choi and rChoi instead of RECIST 1.1 and mRECIST significantly correlated with the OS of patients with uHCC treated with an anti-PD-1 antibody plus bevacizumab. This difference could be explained by several possible reasons. Firstly, the blood supply and hemodynamics of HCC were different from those of mCRC, which might have affected the tumor response to a combined treatment. Secondly, the therapy regimes were not identical for the two types of hepatic malignant tumors.

Previous studies indicated that objective response by mRECIST could serve as a predictor and potential surrogate endpoint of OS in advanced HCC (11, 12, 20). However, in our study, mRECIST was not able to identify the responders from non-responders (*p* = 0.436). This might be explained from two aspects: firstly, the arterial phase can vary for different patients, resulting in the subjective estimation of viable tumors on CT images as assessed by mRECIST, while measurement of the CT density by Choi and rChoi incorporating both viable and non-viable tissues could be done objectively using attenuation values. Secondly, viable lesions defined by mRECIST were applied preferably for the evaluation of intrahepatic lesions; extrahepatic lesions still adhered to RECIST 1.1, but could also respond to treatment and manifest as central necrosis without a decrease in size.

The revised Choi, proposed by Thian et al. based on Choi, has been reported to significantly associate with the OS and PFS of patients with mRCC treated with sunitinib (15). The difference between the Choi and rChoi criteria was mainly the definition of PR. rChoi defined PR as both a decrease of at least 10% in the sum of the diameters of the target lesions and a decrease of 15% in the tumor density, while Choi defined PR as a decrease in either the size or attenuation. In our study, more patients were defined as PR by Choi than by rChoi [30 (51.7%) *vs*. 12 (20.7%)]. The cumulative 1- and 2-year OS rates in responders were 93.3% and 79.8% using Choi and 100.0% and 100.0% using rChoi. It appeared that the Choi criteria categorized more patients as responders compared with the three other criteria, and this might benefit more potential patients. Eighteen patients defined as PR by Choi were redefined as SD by rChoi. The rChoi criteria seemed to be stricter with the definition of PR, and none of the patients classified as responders died during the follow-up period. Consequently, the advantage and disadvantage of the Choi and rChoi criteria should be balanced in clinical decision-making.

The timely identification of responders using appropriate tumor response criteria may reduce the unnecessary drug-related toxicity in patients non-responsive to immunotherapy with targeted therapy. In our study, the evaluation of early tumor response by Choi and rChoi significantly correlated with OS for the combined therapy (*p* = 0.002 and 0.006, respectively). This was consistent with Ronot’s study, which reported that Choi appeared more appropriate than RECIST 1.1 in identifying responders, with better survival for patients with advanced HCC treated with sorafenib (9). The alteration of CT attenuation and lower size decrease threshold (10%) defined by Choi and rChoi may lead to a higher sensitivity in the early detection of responders, as reported in previous studies (21, 22). Besides, it might be inferred that the combination of an anti-PD-1 antibody may synergize more anti-angiogenic effects than the size shrinkage in the early treatment phase, which resulted in more obvious drug-induced necrosis and density decrease.

However, a few limitations should be stressed in this study. Firstly, the limited sample size may impede the generalization of our conclusions in a wider population. Secondly, this was a retrospective study with inevitable potential bias. Further prospective studies with a larger sample size are still needed for validation.

## Conclusions

The evaluation of early tumor response using Choi and rChoi instead of RECIST 1.1 and mRECIST significantly correlated with the OS of patients with uHCC treated with an anti-PD-1 antibody plus bevacizumab. Moreover, the patients identified as responders using the Choi and rChoi criteria showed significantly better OS than the non-responders. The conclusions remain to be verified in further prospective studies with a larger sample size.

## Data Availability Statement

The datasets underlying this study are available on request to the corresponding author. Requests to access these datasets should be directed to dr_fengye_ncc@163.com.

## Ethics Statement

This study protocol was reviewed and approved by the Ethics Committee of National Cancer Center/Cancer Hospital, Chinese Academy of Medical Sciences and Peking Union Medical College (approval No. 18-126/1704), and conducted in compliance with the 1975 Declaration of Helsinki, Good Clinical Practice guidelines and local regulatory requirements. Written informed consent was waived by the Institutional Review Board.

## Author Contributions

Study concept and design (YX, YY, LL), acquisition of data (FY, WZ, HZ, APZ), analysis and interpretation of data (YX), drafting of the manuscript (YX), critical revision of the manuscript for important intellectual content (YX, YY, LL, FY), critical funding (XMZ, HZ, LL), administrative, technical, or material support, study supervision (XMZ, HMZ, APZ, FY, WZ, HZ). All authors contributed to the article and approved the submitted version.

## Funding

This study was supported by the National Natural Science Foundation of China (No. 81971589, 81972311, 82141127), the CAMS Innovation Fund for Medical Sciences (CIFMS) (No. 2021-I2M-1-066), the Non-profit Central Research Institution Fund of Chinese Academy of Medical Sciences (No. 2019PT310026), the Youth Project of Beijing Hope Run Special Fund (No. LC2021B17).

## Conflict of Interest

The authors declare that the research was conducted in the absence of any commercial or financial relationships that could be construed as a potential conflict of interest.

## Publisher’s Note

All claims expressed in this article are solely those of the authors and do not necessarily represent those of their affiliated organizations, or those of the publisher, the editors and the reviewers. Any product that may be evaluated in this article, or claim that may be made by its manufacturer, is not guaranteed or endorsed by the publisher.

## Abbreviations

BCLC, Barcelona Clinic Liver Cancer; CE-CT, contrast-enhanced computed tomography; CR, complete response; ECOG PS, Eastern Cooperative Oncology Group Performance Status; GISTs, gastrointestinal stromal tumors; HCC, hepatocellular carcinoma; HR, hazard ratio; HU, Hounsfield unit; IQR, interquartile range; Mcrc, metastatic colorectal cancer; mRCC, metastatic renal cell carcinoma; mRECIST, modified RECIST; OS, overall survival; PD, progressive disease; PD-1, programmed cell death protein-1; PD-L1, programmed cell death ligand-1; PFS, progression-free survival; PR, partial response; rChoi, revised Choi; RECIST 1.1, Response Evaluation Criteria in Solid Tumors, version 1.1; ROI, region of interest; SD, stable disease; SLD, sum of the longest diameters; TACE, transarterial chemoembolization; uHCC, unresectable HCC.
